# Compassionate use of cefiderocol for carbapenem-resistant *Acinetobacter baumannii* prosthetic joint infection

**DOI:** 10.1093/jacamr/dlab055

**Published:** 2021-06-15

**Authors:** Diana A Mabayoje, Caoimhe NicFhogartaigh, Benny P Cherian, Mei Gie Meiqi Tan, David W Wareham

**Affiliations:** 1 Division of Infection, Barts Health NHS Trust, London, UK; 2 Antimicrobial Research Group, Blizard Institute, Queen Mary University of London, London, UK

## Abstract

**Background:**

Cefiderocol is a recently licensed novel siderophore-conjugated cephalosporin stable to hydrolysis by serine and MBLs. It has been successfully used to treat Enterobacterales infections and is approved for the treatment of infections due to aerobic Gram-negative organisms in adults with limited treatment options.

**Objectives:**

To describe the compassionate use of cefiderocol and clinical outcome in a case of prosthetic joint infection due to MDR *Acinetobacter baumannii*.

**Patients and methods:**

This case study follows a 66-year-old woman who sustained an open fracture of the left distal humerus in Pakistan. She underwent open reduction and internal fixation and on return to the UK presented to hospital with a discharging surgical wound.

**Results:**

Debridement of her wound cultured NDM carbapenemase-producing *A. baumannii* susceptible to colistin, tobramycin and tigecycline only. She developed vomiting with acute kidney injury with colistin and tigecycline. Antimicrobial efficacy of cefiderocol was predicted from *in vitro* and *in vivo* susceptibility tests. A successful request was made to Shionogi for compassionate use of cefiderocol, which was added to tigecycline. Cefiderocol was well tolerated with no toxicity and improved renal function. In total she received 25 days of cefiderocol and continued on tigecycline for a further 6 weeks in the community. She has well-healed wounds and good range of elbow movement.

**Conclusions:**

Cefiderocol’s novel mode of cell entry is effective against MDR Gram-negative bacteria with reduced toxicity compared with other last line antibiotics. Our case demonstrates that cefiderocol may be useful as therapy for patients with limited treatment options due to antimicrobial resistance. **The prescribing information for cefiderocol is available at: https://shionogi-eu-content.com/gb/fetcroja/pi.**

## Patient and methods

**Figure 1.**
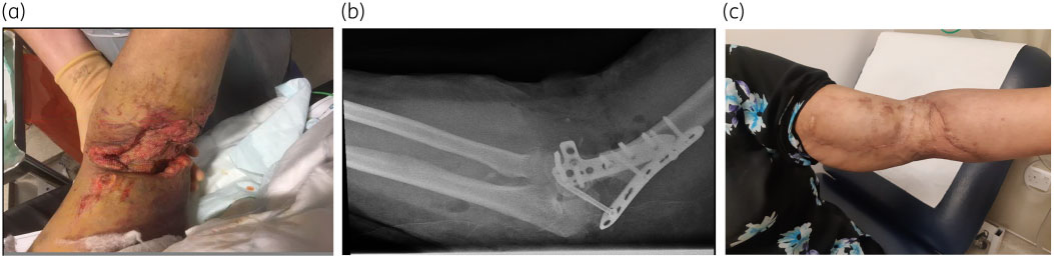
(a) Open wound in left arm at time of presentation. (b) Plain X-ray of left arm at time of presentation. (c) Left elbow after completion of antimicrobial therapy.

A 66-year-old female with a past medical history of hypothyroidism and hypertension sustained an open fracture of the left distal humerus following a road traffic accident in Pakistan. She underwent open reduction and internal fixation (ORIF) in Pakistan, and upon return to the UK presented immediately to hospital with a discharging surgical wound (Figure 1a). On initial examination she was haemodynamically stable with normal physical parameters, inflammatory markers were slightly elevated—WBC count 10.2 × 10^9^ cells/L, C-reactive protein (CRP) of 65 mg/L—but renal and liver function were normal. Admission screening (selective bacterial culture) for MRSA and carbapenem-resistant organisms (CRO) was negative. Radiological imaging of the left arm showed an existing comminuted fracture of the distal left humerus managed with internal fixation (Figure 1b). There were multiple locules of subcutaneous gas and soft tissue stranding extending from the level of the mid/distal humerus to the mid forearm, suggestive of active infection. The patient underwent washout, surgical debridement and removal of metalwork on Day 3. Due to instability of the elbow, the humeral plate was left *in situ* and an external fixator applied. Meropenem and teicoplanin were commenced as empirical therapy antimicrobial pending microbiological results. Intra-operative samples subsequently cultured carbapenem-resistant *Acinetobacter baumannii* (AB4 2019) susceptible only to polymyxins, tobramycin and tigecycline on routine antimicrobial susceptibility testing (Table 1).

**Table 1.** Phenotypic (MicroScan WalkAway System) and genotypic (Illumina WGS) resistance in *A. baumannii* AB4

Antimicrobial/Resistance determinants	MIC (mg/L)							
β*-*lactams	AMP	AMC	ATM	CAZ	CTX	CZA	CXM	DOR
* bla*_NDM-1_, *bla*_OXA-23_, *bla*_OXA-69_, *bla*_ADC-25_	>256	>8	>4	>256	>64	>256	>256	>256
	ETP	FEP	FOX	IPM	IMR	MEM	TZP	SUL
	>1	>16	>256	>32	>32	>32	>64	>4
Aminoglycosides	AMK	GEN	TOB					
* aac(3’)-Ia/aadA, aph(3')-Ia, aph(3’)-Ib/straA, aph(3’)-VIa, aph(6)-Id/straB*	>16	>4	**<2**					
Quinolones	CIP	LVX	MXF					
	>1	>1	>1					
Tetracyclines	TET	TGC						
*tet*(B)	>256	**2**						
Sulphonamides/trimethoprim	SXT	TMP						
* dfrA1, sul1, sul2*	>4	>256						
Polymyxins	CST	PMB						
	**0.5**	**0.5**						
Others (macrolides, chloramphenicol, fosfomycin)	AZM	CLI	CHL	ERY	FOF			
	>256	>256	>256	>256	64			

Bold formatting indicates susceptibility.

AMP, ampicillin; AMC, amoxicillin/clavulanate; ATM, aztreonam; CAZ, ceftazidime; CTX, cefotaxime; CZA, ceftazidime/avibactam; CXM, cefuroxime; DOR, doripenem; ETP, ertapenem; FEP, cefepime; FOX, cefoxitin; IPM, imipenem; IMR, imipenem/relebactam; MEM, meropenem; TZP, piperacillin/tazobactam; SUL, sulbactam; AMK, amikacin; GEN, gentamicin; TOB, tobramycin; CIP, ciprofloxacin; LVX, levofloxacin; MXF, moxifloxacin; TET, tetracycline; TGC, tigecycline; SXT, trimethoprim/sulfamethoxazole; TMP, trimethoprim; CST, colistin; PMB, polymyxin B; AZM, azithromycin; CLI, clindamycin; CHL, chloramphenicol; ERY, erythromycin; FOF, fosfomycin.

WGS (Illumina, MiSeq) was used to characterize the genotype and resistance determinants. This identified the isolate as *A. baumannii* ST1 in the Pasteur MLST scheme and as ST231 in the Oxford scheme (https://pubmlst.org/abaumannii/). Both typing schemes identified the isolate as a member of International Clone I (IC1).^1^ AB4 2019 carried multiple genes conferring resistance to all β-lactams (*bla*_NDM-1_, *bla*_OXA-23_, *bla*_OXA-69_, *bla*_ADC-25_), aminoglycosides (*aac(3’)-Ia/aadA, aph(3')-Ia, aph(3’)-Ib/straA, aph(3’)-VIa, aph(6)-Id/straB*), quinolones, trimethoprim and sulphonamides (*dfrA1, sul1, sul2*).

On Day 10 the patient underwent further debridement, insertion of aminoglycoside impregnated beads and due to worsening signs of infection antibiotic therapy was switched to IV colistin methanesulphonate (9 MU loading dose followed by 3 MU 8 hourly) and tigecycline (100 mg and then 50 mg 12 hourly). She then underwent left distal humerus revision and medial collateral ligament reconstruction on Day 17 of admission. Further intra-operative samples isolated the same organism both by culture and direct 16S rDNA PCR sequencing of the tissue. Five days into treatment with colistin and tigecycline she developed severe vomiting, dehydration and acute kidney injury [creatinine 440 μmol/L from a baseline of 80 μmol/L, accompanied by high trough (4.6 mg/L) and random (6.6 mg/L) serum colistin (recommended pre-dose range 2–4 mg/L)]. Colistin therapy was therefore discontinued, and she was given IV fluids to correct her acute kidney injury and antiemetics for symptomatic control. Tigecycline was continued due to the lack of alternative therapeutic options however her inflammatory markers continued to rise with a peak CRP of 188 mg/L and a WBC count of 13.4 × 10^9^ cells/L (neutrophils 10.4 × 10^9^ cells/L).

A written request was thus made to Shionogi via the compassionate use programme for treatment with cefiderocol. This was approved and cefiderocol started on Day 36 of admission on a renally adjusted dose of 0.75 g 12 hourly. As renal function improved, the dose of cefiderocol dose was increased to 1.5 g thrice daily and her gastrointestinal upset improved with symptomatic treatment. The CRP level declined from 188 mg/L to 3  mg/L, and she underwent successful skin grafting and removal of the external fixator. In total she received 25 days of cefiderocol and then continued on tigecycline (50 mg 12 hourly) for a further 6 weeks in the community. At the end of cefiderocol therapy her renal function had normalized (creatinine 73 μmol/L) and no adverse effects were noted. She had well-healed wounds and good range of elbow movement following completion of antibiotic treatment (Figure 1c).

**Figure 2.**
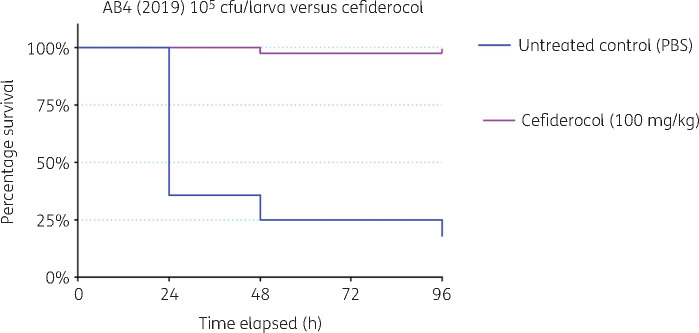
Efficacy of cefiderocol versus *A. baumannii* infection in *Galleria mellonella.* Survival curve over 96 h (Kaplan–Meier *P *<* *0.05).

The activity and efficacy of cefiderocol against the *A. baumannii* (AB4 2019) isolates was assessed *in vitro* and *in vivo* using an invertebrate model of *A. baumannii* infection.^2^ Susceptibility testing by disc diffusion using 30 μg cefiderocol discs on un-supplemented Mueller–Hinton II agar gave zones of inhibition of 17 mm. The MIC of cefiderocol obtained by broth microtitre dilution using Sensititre CMP1SHIS plates (Themo Scientific, Basingstoke) supplied for evaluation was 4 mg/L. Insufficient evidence is currently unavailable for EUCAST breakpoints to be set for susceptibility of *Acinetobacter* spp. to cefiderocol, although a zone diameter of ≤17 mm and an MIC of ≤2 mg/L have been proposed as non-species specific PK/PD breakpoints.^3^ Cefiderocol administered at a humanized dose of 100 mg/kg was 95% effective in the treatment of *Galleria mellonella* infected with a lethal dose (10^5^ cfu/larva) of *A. baumannii* AB4 2019. No toxicity of cefiderocol was observed in *G. mellonella* across an escalating dose range of 10–100 mg/kg (Figure 2).

### Ethics

Ethics approval was not required for this study; we obtained written informed consent from the patient. Photographs were taken with informed consent from the patient.

## Discussion

*A. baumannii* is an important nosocomial pathogen that frequently exhibits multidrug resistance. It is a recognized cause of hospital- and ventilator-associated pneumonia, catheter-related bloodstream infections and also traumatic surgical wound and prosthetic device infections.^4^^,^^5^ The emergence and spread of *A. baumannii* worldwide as WHO critical priority pathogen has been facilitated by the organism’s ability to acquire resistance genes and the success of a number of international clones.^6^ This patient likely acquired their infection following healthcare contact in Pakistan, a country with high rates of nosocomially acquired carbapenem-resistant Gram-negatives.^7^ In studies from tertiary care hospitals in Lahore, MDR *A. baumannii* was isolated in up to 31% of pus samples and 17.5% of blood cultures^8^ with the commonest mechanisms of carbapenem resistance being production of OXA-51, OXA-23 and NDM-1 carbapenemases. Amongst existing drugs, the most effective *in vitro* were tigecycline (80% susceptibility) among isolates, followed by colistin with 50% susceptibility.^9^

The case demonstrates that treatment of MDR *A. baumannii* with last line antibiotics such as tigecycline and colistin is associated with significant toxicity issues and the need for therapeutic drug monitoring. Infected metalwork requires prolonged antibiotic therapy and our patient suffered gastrointestinal disturbance with tigecycline and severe nephrotoxicity due to colistin.

Cefiderocol is a novel antibiotic that utilizes bacterial iron uptake pathways to bypass Gram-negative resistance mechanisms. The addition of a catechol moiety binds ferric iron, an essential nutrient for bacterial growth that facilitates active transport of the molecule across the outer membrane of Gram-negative bacteria.^10^ The β-lactam backbone combines the active residues of ceftazidime and cefepime, rendering the molecule stable to hydrolysis by most serine- and metallo-β-lactamases. Cefiderocol has been shown to have activity against carbapenem-resistant Gram-negative bacteria including lactose nonfermenters.^11–13^ Most studies involving cefiderocol have been in complicated urinary tract infections and hospital-acquired pneumonia, however, it has been granted license in the EU for management of resistant Gram-negative infections in any site.^14–16^ In infections at deep sites or implant-associated infection a longer course is required in combination with source control.^17^^,^^18^ Our experience with cefiderocol was more successful than the findings in the CREDIBLE-CR study, which demonstrated a higher rate of clinical failure and increased mortality in patients with resistant *A. baumannii* infection receiving cefiderocol versus best available therapy.^19^ Other case reports have also demonstrated efficacious use of cefiderocol in MDR infections, including complicated bone and *A. baumannii* joint infections.^20^^,^^21^ Antimicrobial efficacy of cefiderocol against the MDR isolate in our case was predicted by *in vitro* and *in vivo* susceptibility tests. As demonstrated by our case, cefiderocol may be useful as salvage therapy for patients with limited treatment options due to resistance or drug-related toxicity.

## Funding

This study was carried out as part of our routine clinical work and supported by funding from Queen Mary University of London.

## Transparency declarations

D.W.W. has served on advisory boards for Shionogi, Pfizer and Merck. All other authors: none to declare. This article is part of a promotional Supplement developed and sponsored by Shionogi B.V. The accompanying video formed part of an educational webinar for which the author received a speaking honorarium. The material underwent peer review by the Supplement Editors. Editorial assistance to Shionogi Europe was provided by Page Medical.

## Supplementary data

The video transcript is available as Supplementary data at *JAC-AMR* Online.

## Supplementary Material

dlab055_Supplementary_Data
