# Supplementary material for: Compassionate use of cefiderocol for carbapenem-resistant Acinetobacter baumannii prosthetic joint infection
Source: JAC Antimicrob Resist. 2021 Jun 15;3(Suppl 1):i21–4. doi: 10.1093/jacamr/dlab055 (PMC8251250; doi:10.1093/jacamr/dlab055)
Supplement: dlab055_Supplementary_Data [file dlab055_Supplementary_Data.docx]

**Supplementary data**

**Video transcript**

I would like to present a case of compassionate use of cefiderocol for carbapenem-resistant *Acinetobacter baumannii* prosthetic joint infection. My name is David Wareham, and I am a microbiologist. Although I was barely involved with this, it was really down to the good work of my colleagues here and almost certainly the orthopaedic surgeons in getting a good outcome in this case. These are disclosures or any conflicts that I might have.

The patient, first of all, was a 66-year-old lady who had been on holiday in Pakistan. Unfortunately, while she was there, she had a road traffic accident. She was hit by a vehicle and broke her arm, sustaining an open fracture to her left distal humerus. She was therefore transferred to a hospital in Lahore where she was treated and underwent open reduction of that fracture. She went back to theatre several times in the hospital in Pakistan, but the wound was left open and not closed and she was discharged with this open wound. She immediately flew back to the UK and when she arrived here, she presented immediately to a hospital with this—which clearly was a rather severe infection of the elbow and the soft tissues surrounding it.

You can see from the X-ray that there is metal work in there, which looks quite displaced, pins, and there is a lot of gas in the soft tissues, consistent with infection within that area. So, she was admitted. This was washed out and debrided by the surgeons. And, probably quite wisely, she was commenced on very broad-spectrum antibiotics—meropenem and teicoplanin. Tissue samples were taken from the initial debridement which were *Acinetobacter baumannii*, which you can see here—and which was multidrug resistant. It was resistant to virtually everything we tested apart from aminoglycosides and colistin and tigecycline.

That isolate was sequenced and turned out to be one of these very extensively drug-resistant strains of *Acinetobacter baumannii*, with all of the genes which give resistance to most classes of antibiotics including β-lactams like OXA-23, which I talked about before, and also NDM-1. So multiple carbapenemases, with resistance genes giving it resistance to aminoglycosides and anything else you might want to use against it.

This wound continued to discharge, and she had a vacuum dressing put upon it. She initially had a rather stormy course and the inflammatory markers rose. CRP went up to 140 and she had more pain around the arm. Having had the results of the cultures back, the meropenem and glycopeptide was changed to colistin and tigecycline. She got nausea and vomiting whilst on that, underwent more surgery about a week later, and we continually grow the same organism from the pus.

This shows her renal function, which became a problem when the colistin and tigecycline commenced. Within several days she had very severe deterioration: renal function, acute kidney injury, creatinine going from normal to more than 400 and that was associated with very high serial trough colistin levels way above the recommended level of 2–4 mg/L. So that ensured that we had to stop the colistin.

We therefore had to consider something else. At this point, the decision was made to give compassionate use of cefiderocol and that was approved. That was commenced at a low dose of 0.75 g 12 hourly, infused over 3 h. Tigecycline was continued. The renal function improved a bit, and they increased the cefiderocol dose to 1 g 8 hourly. The isolate was deemed to be susceptible to cefiderocol by this diffusion borderline—17.5 mm on the disc susceptibility test and an MIC of 4 mg/L. Again, as renal function improved, we increased the dose to 1.5 g three times a day eventually. She received that for about another 2 weeks. The tigecycline, however, remained at a lower dose of 15 mg 12 hourly for another 2 weeks following the cessation of the cefiderocol.

We checked whether the isolate was actually susceptible to cefiderocol in an *in vivo* model. We use a model which looks at activity in a larvae model of infection. You can see that cefiderocol here in the infected larvae is completely effective compared to no treatment at all.

This is to show you the improvement, and the time course you would use on the antibiotics. Initially, the patient was very unwell, and CRP rose. Cefiderocol was commenced and tigecycline was commenced, and gradually returned to normal. Likewise, white cell count did come back to normal and remained at normal after treatment.

That is her arm after she was treated for nearly 6 weeks with these antibiotics. I would say that she responded well to cefiderocol treatment for the joint infection that was affected by toxicity from colistin.
